# Strategies to support women in research publishing

**DOI:** 10.1038/s44172-023-00090-3

**Published:** 2023-06-23

## Abstract

On International Women in Engineering Day, we outline the ways that *Communications Engineering* supports the equitable inclusion of women in our publishing activities. We also suggest ways in which researchers can be supporters of diversity and inclusion when publishing their own research.

On 23rd June, *Communications Engineering* celebrates International Women in Engineering Day (INWED). Women continue to be under-represented in engineering and engineering research. We are heartened to see an ever-increasing number of initiatives across the globe that encourage women into engineering degrees and academic positions. However, we must not only take steps to create a diverse research community. We must also ensure that this diversity is harnessed to its fullest, and with everyone having the same opportunity to contribute.

Our parent company, Springer Nature, has for some time had a formal commitment in place to accelerate diversity and inclusion. Since 2019, the group has committed to intentionally and pro-actively reach out to women researchers when commissioning externally authored content, recruiting peer reviewers, and inviting event speakers (https://www.springernature.com/gp/advancing-discovery/springboard/blog/new-diversity-commitment/17485502). *Communications Engineering* is a willing participant in these commitments. We also encourage all researchers to include women co-authors when building their author teams for the writing of non-primary content. It is reassuring that we often receive responses that reflect the same desire for gender balanced authorship.

Springer Nature has also taken some steps to encourage team leaders and recruiters to consider balance in their editorial teams (https://www.springernature.com/gp/researchers/the-source/blog/blogposts-for-peer-reviewers/diversity-inclusion-peer-reviewer-editorial-recruitment/23486026). This has been a key goal of *Communications Engineering*, and we are delighted that at the time of writing, *Communications Engineering* has a 50:50 balance of women and men in our editorial board. We also aim to have at least one woman in all our guest editor panels for all our Collections.

We will only know if our practices have any impact on authorship if we collect data on the gender of our authors. In March 2023 Springer Nature began its rollout of the collection of self-reported data on the gender of our authors, which has been implemented on the manuscript tracking systems of a select number of journals (https://www.springernature.com/gp/researchers/the-source/blog/blogposts-life-in-research/the-need-for-data-in-the-path-to-gender-equity-in-publishing/24089968). The intention is to slowly scale this activity until it is active across all journals. *Communications Engineering* has been part of this project since late April. When authors submit a manuscript, they are asked for details on gender; the options are man, woman, non-binary or gender diverse, and prefer not to disclose. In this way we join other publishers in observing the impact of new initiatives on representation and charting the progress of gender equity.

In the publishing space, there are some steps in which researchers as authors can support women. First, the recommended reviewer lists that we see in cover letters which support submissions often contain entirely men. We encourage you to also suggest women in your recommended reviewer lists. Tell us about the female rising stars, fantastic postdocs and assistant professors, as well as more established academics. Second, consider your reference lists. There are now numerous studies that show that across a variety of research fields, women are not cited as much as their male counterparts^1^. In these days where so much weight is put on citation metrics, consider carefully to whom you are allocating your privileged citation opportunities. Finally, to PIs: where it is appropriate, take care to ensure that the women who contributed to the research get recognised via authorship^2^. Even more powerful is to (again where appropriate) actively encourage the women in your group to be first and/or corresponding authors. The truth is that these are the names that others will look at and remember the most, from editors, to peer reviewers, to readers.

In recognition of INWED, today we publish two interesting papers that have a women-to-men author balance of 50:50 or greater. For primary research this is rare in our pages. First, we publish a paper from Kristen Brosamer, Katerina Kourentzi, Richard C. Willson and Binh V. Vu describing a sensitive lateral flow assay platform using the chemistry of glowsticks to report the detection of human chorionic gonadotropin and SARS-CoV-2 nucleoprotein, read by an unmodified smartphone. The researchers also provide evidence that the platform could offer multiplexed detection of analytes on a single test line^3^. In a second paper, Kai Junge, Catarina Pires and Josie Hughes describe a sensorized physical simulator of a real raspberry plant (i.e. a physical twin), and demonstrate that its use in lab-based training is sufficient for direct lab to field transfer of a robotic raspberry picker^4^.

Our journal is only a tiny part of the picture. We thank the many other men and women who work hard to create equal opportunities in different settings, from schools to higher education institutes and corporate settings. We also thank those who recognise and support women through additional life challenges such as those with caring responsibilities and those who are coping with menopause, to enable them to continue to make significant contributions in the workplace. Keeping diversity, equity and inclusion at the forefront of our minds will lead to diverse teams of the future creating exciting technologies and engineering solutions such as those described above, that will benefit us all.
